# Inhibition of peritoneal dissemination of colon cancer by hyperthermic CO2 insufflation: A novel approach to prevent intraperitoneal tumor spread

**DOI:** 10.1371/journal.pone.0172097

**Published:** 2017-02-16

**Authors:** Yuanfei Peng, Hua Yang, Qing Ye, Houming Zhou, Minhua Zheng, Yinghong Shi

**Affiliations:** 1 Department of Liver Surgery, Liver Cancer Institute, Zhongshan Hospital, Fudan University; Key Laboratory of Carcinogenesis and Cancer Invasion of Ministry of Education, Shanghai, China; 2 Department of Gastrointestinal Tumor Surgery, Fujian Provincial Tumor Hospital of Fujian Medical University, Fujian, China; 3 Department of General Surgery, Shanghai Minimally Invasive Surgery Center, Institute of Digestive Surgery, Ruijin Hospital, Shanghai Jiaotong University School of Medicine, Shanghai, China; German Cancer Research Center (DKFZ), GERMANY

## Abstract

**Background:**

The increasing use of laparoscopic surgery for advanced gastrointestinal cancer raises concerns about intra-peritoneal tumor spread. Prevention of peritoneal dissemination is extremely important but a preventive modality is lacking. The aim of this study was to examine a novel approach (hyperthermic CO2 insufflation, HT-CO2) for preventing peritoneal dissemination during laparoscopic surgery.

**Methods:**

A peritoneal dissemination model was established in Balb/c nu/nu mice by intraperitoneal injection of human colon cancer cells (SW1116, 1×10^6^). The mice (n = 48) were subsequently randomized into two groups and subjected to hyperthermic CO2 (43°C, >95% humidity, HT-CO2 group) or standard normothermic CO2 (21°C, <1% relative humidity, NT-CO2 group) insufflation for 3 hours. The mice were sacrificed 28 days later. The peritoneal dissemination was quantitatively analyzed by counting and weighing the peritoneal nodules. The port sites and ascites volume were measured. The peritoneal damage of HT-CO2 was histologically examined with light microscopy and scanning electron microscopy. Intra-abdominal adhesions were evaluated 4 weeks later.

**Results:**

The number of peritoneal nodules in the HT-CO2 group was significantly less than that in the NT-CO2 group (10.21±3.72 vs. 67.12±5.49, *P*<0.01). The mean weight of metastatic tumors in the HT-CO2 group was significantly lower than that in the NT-CO2 group (0.31±0.10g vs. 2.16±0.31g, *P*<0.01). Massive ascites were found in the NT-CO2 group while significantly less ascites developed in HT-CO2- treated mice (8.26±0.31ml vs. 1.27±0.28ml, *P*<0.01). No port-site metastases were detected in the HT-CO2 group while the incidence of the NT-CO2 group was 12.5% (3/24). HT-CO2 subjection resulted in slight peritoneal damage; the peritoneum returned to normal within five days. No adhesions formed after HT-CO2 treatment.

**Conclusions:**

HT-CO2 can suppress peritoneal dissemination of colon cancer cells and only causes slight and transient peritoneal damage. HT-CO2 may serve as a promising adjuvant treatment for preventing peritoneal dissemination in laparoscopic resection of advanced colorectal cancer.

## Introduction

Minimally invasive techniques are becoming increasingly common for the treatment of advanced gastrointestinal cancer. There is growing concern that such treatments may increase the likelihood of peritoneal dissemination. Peritoneal dissemination frequently occurs during the surgical removal of advanced gastrointestinal cancer. During laparoscopic surgery liberation of cells from the primary tumor and intraoperative spillage of tumor cells may occur. The liberation of cells from the primary tumor is a critical event in peritoneal dissemination and port-site metastasis [1]. The insufflated CO2 can promote the peritoneal dissemination of the spilled tumor cells [2]. Diffuse damage of the entire peritoneum often takes place after CO2 pneumoperitoneum [3]. One of our previous studies shows that prolonged standard CO2 insufflation leads to serious damages of the peritoneum [4]. The peritoneal damage provoked by the pneumoperitoneum can induce a specific intraperitoneal tumor implantation [3]. Tumor cells are able to attach to the denuded basal lamina and form predominantly diffuse metastases throughout the peritoneum within 96 hours after pneumoperitoneum [3]. A large number of experimental studies have demonstrated that laparoscopic cancer surgery with CO2 insufflation is associated with a significant increase in the incidence of wider dissemination and implantation of intra-abdominal tumor, as well as port-site metastases [5]. In laparoscopic surgery for advanced gastrointestinal cancer the incidence of peritoneal dissemination and port-site metastases is thought to be much higher.

Peritoneal carcinomatosis is associated with poor prognosis as it responds poorly to current treatments [6]. It poses a serious challenge and prevention is extremely important. However, preventive modalities are still lacking, especially for laparoscopic surgery. Developing an effective approach to prevent peritoneal dissemination for laparoscopic surgery is urgently required.

In our previous study we attempted a newly developed approach (hyperthermic CO2 insufflation, HT-CO2). The CO2 used for pneumoperitoneum was heated to hyperthermic temperature (43–44°C) to kill the spilled colorectal cancer cells generated during laparoscopic cancer surgery and therefore prevent or suppress peritoneal dissemination. Our previous in vitro study showed that HT-CO2 (43–44°C for 2–4 h) has a significant cytotoxic effect on colon and gastric cancer cells, suggesting that HT-CO2 may serve as a new approach for preventing peritoneal dissemination of laparoscopic gastrointestinal cancer surgery [7,8].

The aim of this study was to examine whether HT-CO2 insufflation could prevent intra-peritoneal tumor spread in vivo. A peritoneal dissemination model was established by using a cell inoculation model which mimics the tumor spillage during laparoscopic surgery. The preventive effect of HT-CO2 on the peritoneal dissemination of colon cells was examined. Meanwhile, the effect of HT-CO2 on the peritoneum and whether HT-CO2 causes peritoneal damage were also evaluated.

## Materials and methods

### Cell lines and animals

Human colon cancer cell lines SW1116 (ATCC) were grown in RPMI1640 supplemented with 10% FBS at 37°C in humidified air containing 5% CO2.

Male BALB/c nu/nu mice (6 weeks old, Chinese Academy of Science) were bred in specific pathogen-free conditions. All the animals were cared for and handled according to the Guide for the Care and Use of Laboratory Animals published by the National Institutes of Health. Experimental protocol was approved by Shanghai Medical Experimental Animal Care Committee. All efforts were made to minimize perioperative suffering (S1 File).

### Experimental design of gas insufflation

**Fig 1:**
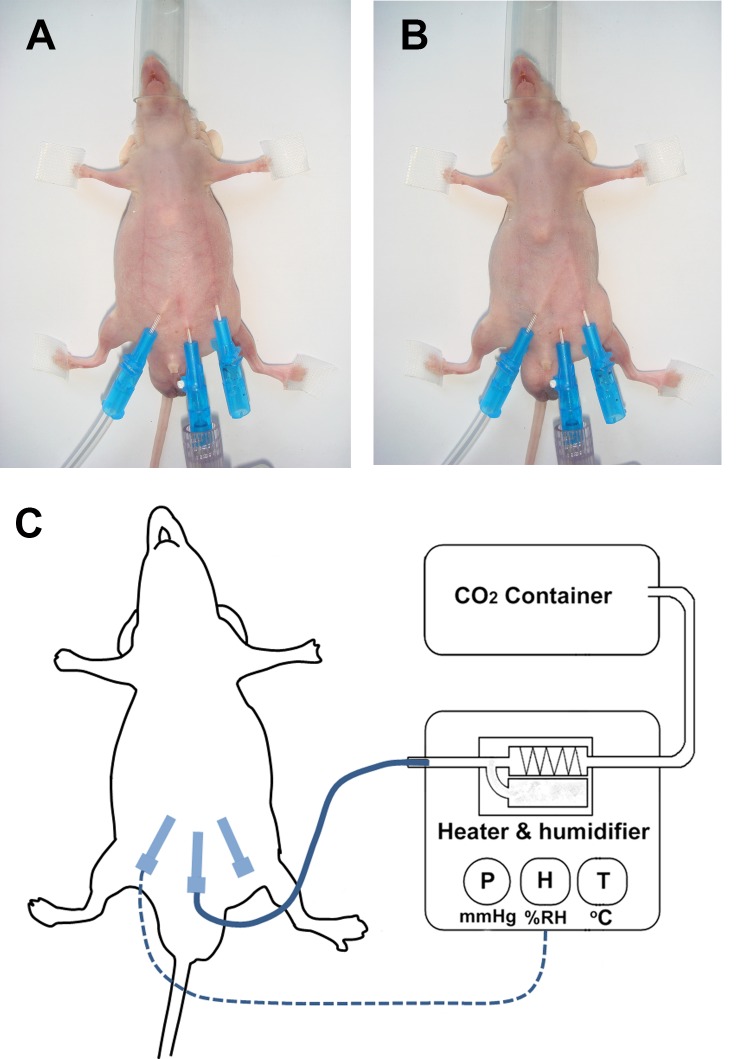
Experimental setup. A G22 intravenous cannula is inserted into the lower midline and serves as the first trocar. Another G22 intravenous cannula is inserted into the right lower quadrant and used for measurement of intraperitoneal pressure, humidity and temperature. The hyperthermic CO2 (HT-CO2) or normothermic CO2 (NT-CO2) is generated by a CO2 heater & humidifier device and insufflated into the peritoneal cavity of the mouse. The intra-abdominal temperature (T), relative humidity (H) and pressure (P) are monitored in a real-time manner and kept constant.

For evaluation of the effect of HT-CO2 on peritoneal dissemination or peritoneal damage, the Balb/c nu/nu mice were randomly allocated to two groups: the HT-CO2 group and the NT-CO2 group. The mice in the HT-CO2 group (n = 24) and NT-CO2 group (n = 24) were subjected to HT-CO2 (hyperthermic CO2, 43°C, 95% relative humidity) and NT-CO2 (standard normothermic CO2, 21°C, <1% relative humidity) insufflations for 3h, respectively. All surgical procedures were performed under sterile conditions. The operation procedures for CO2 insufflation were as follows. All mice were anaesthetized in a container with 3–5% vaporized isoflurane with air. Each spontaneously breathing, anaesthetized animal was placed in the supine position on a surgical warming table. Anesthesia was maintained by delivering 3–5% vaporized isoflurane with air through a nose cone. After cleansing with 70% alcohol, the abdominal wall of the mouse was lifted and a 22-gauge intravenous catheter (Vasocan, Braun, Germany) was inserted into the lower midline of the abdomen and connected to the CO2 insufflator. The peritoneal space was insufflated with CO2 to a pressure of 6 mmHg. After establishment of pneumoperitoneum, two other needles were inserted into the left lower quadrant and the right lower quadrant, respectively. They were correspondingly connected to pressure, humidity and thermal detectors (Fig 1). The HT-CO2 or NT-CO2 was insufflated into the peritoneal cavity at 6 mmHg with a flow rate of 0.5 L/min. The HT-CO2 and NT-CO2 were provided by a CO2 heater and humidifier device (patent protected by State Intellectual Property Office of China, No. 2006200477736) as previously described [4]. The device was equipped with a control system which regulated the temperature (T), relative humidity (H) and pressure (P) of the gas insufflated into the peritoneal cavity to guarantee their high stability. The gas was completely exsufflated at the end of the procedure. Port site wounds were not closed in any of the animals after treatment. Post-operative analgesics were used for all mice undergoing surgery (0.25% bupivicaine drops at the port sites + 1.6mg/ml acetaminophen-treated drinking water for 48 hours post-surgically).

### Evaluation of the effect of HT-CO2 on peritoneal dissemination of colon cancer cells in vivo

A Cell inoculation model was used to examine the preventive effect of HT-CO2 on peritoneal dissemination. Experimental peritoneal tumor spread was induced by intraperitoneal injection of human colon cancer cells. The HT-CO2 was insufflated into the peritoneal cavity after inoculation of the tumor cells and the effect of HT-CO2 on human colon cancer cells was examined 28 days later. Briefly, a total of 48 mice were randomly allocated to the HT-CO2 and NT-CO2 groups. Cannulas were inserted as described above. The human colon cancer cells (SW1116) were harvested, washed and suspended in phosphate buffered saline. All mice were intraperitoneally injected with 1×10^6^ SW1116 cells via the inserted cannula to simulate tumor cells spillage during laparoscopic colon cancer surgery. The abdomen was massaged to disperse the tumor cells. The HT-CO2 or NT-CO2 was then insufflated into the peritoneal cavity at 6 mmHg and 0.5L/min for 3 hours as described above. The port sites were not closed after the pneumoperitoneum was removed. All mice were housed in a specific pathogen-free barrier facility and observed twice a day after the procedure. At day 28 after tumor cell inoculation all mice were sacrificed. Euthanasia by cervical dislocation was used to sacrifice mice after the mice were anaesthetized in a container with 3–5% vaporized isoflurane with air. The peritoneal tumor spread was assessed in a blinded manner by two independent histopathologists. The number of peritoneal and serosal nodules was counted. Then the tumor nodules were excised and weighed. The port site metastasis was determined as follow. The port sites were marked after the CO2 insufflation and recorded daily to ensure the location of the port sites. After 28 days the peritoneum corresponding to the port sites were examined and the port site metastasis was determined. The ascites were quantified by measuring the volume of ascites.

### Evaluation of the effect of HT-CO2 on peritoneal damages

A total of 50 mice were randomly allocated to two groups: the HT-CO2 group and the NT-CO2 group (25 mice for each group). The mice were subjected to HT-CO2 or NT-CO2 insufflation for 3 hours as described above. In each group, five mice were sacrificed at 6, 24, 48 or 96 h after treatment. Euthanasia by cervical dislocation was used to sacrifice mice after the mice were anaesthetized in a container with 3–5% vaporized isoflurane with air. The peritoneal injury was analyzed histologically by an independent observer who was blind to the experimental design. The light microscopic analysis and scanning electron microscopy analysis were performed as previously described [4]. The other 5 mice in each group were killed for analysis of intra-abdominal adhesions at 4 weeks after treatment. The peritoneum was examined and the adhesions were scored as previously described [4]. The mice in the NT-CO2 group served as controls for analysis of peritoneal damages.

### Statistical analysis

Statistical analyses were performed with SPSS software (SPSS version 22, IBM Corp., USA). Results were presented as means±SD. Differences between groups were evaluated with a student t-test. Differences between groups were considered statistically significant at *P* < 0.05.

## Results

All animals survived the study. There were no differences among all groups in body weight and induction of anesthesia. All mice tolerated the HT-CO2 or NT-CO2 insufflation.

### HT-CO2 significantly suppresses peritoneal dissemination

**Fig 2:**
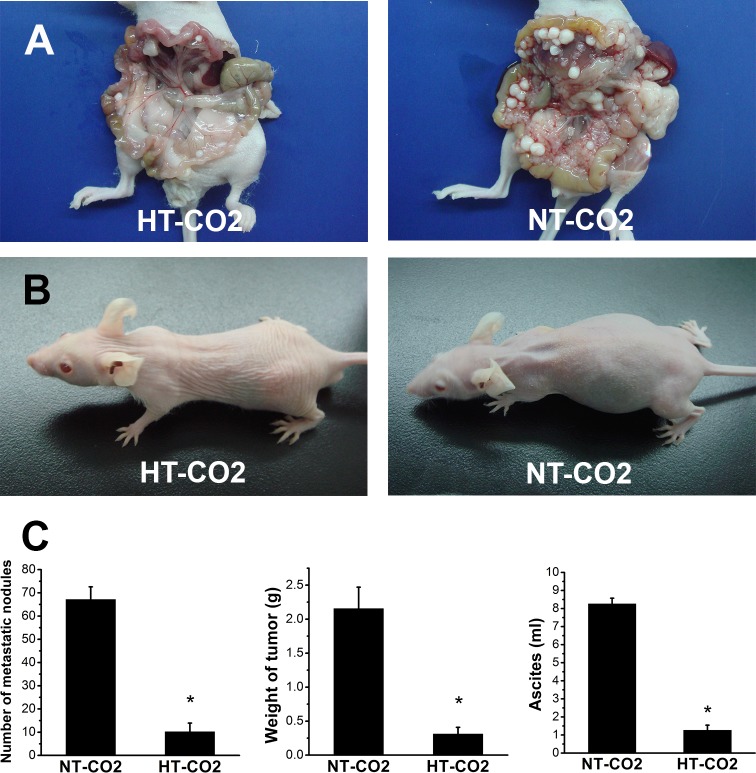
HT-CO2 significantly suppresses peritoneal dissemination and ascites formation. (**A**) Macroscopic view of the intraperitoneal tumor spread. Metastatic tumor nodules can be seen growing on the mesenterium, peritoneum, greater omentum, diaphragm and the surface of the liver. The number of metastatic nodules in mice receiving HT-CO2 insufflation is significantly less than that in mice subjected to NT-CO2. (**B**) Ascites of mice receiving HT-CO2 treatments are significantly less than those subjected to NT-CO2 insufflation. (**C**) Statistical analysis of the number of peritoneal metastasis, weight of tumor nodule and ascites volume. Data are presented as means ± SD; *: *P*<0.01, HT-CO2 *vs* NT-CO2.

The intraperitoneal metastases occurred in both the HT-CO2 and NT-CO2 groups. However, the number of peritoneal nodules in the HT-CO2 group was significantly less than that in the NT-CO2 group. The mean number of peritoneal and serosal nodules in the HT-CO2 group was 10.21±3.72 while that in the NT-CO2 was 67.12±5.49 (*P*<0.01) (Fig 2). Additionally, the size of nodules in the HT-CO2 group was significantly smaller than that in the NT-CO2 group (Fig 2). The weight of tumor nodules in the HT-CO2 group was significantly lower than that in NT-CO2 group (*P*<0.01). The mean weight of tumor nodules in mice receiving HT-CO2 was 0.31±0.10g while that in mice subjected to NT-CO2 was 2.16±0.31g. Massive ascites were found in the NT-CO2 group. In contrast, less ascites developed in HT-CO2- treated mice. The mean volume of ascites was 1.27±0.28ml in the HT-CO2 group and 8.26±0.31ml in the NT-CO2 group (*P*<0.01). Therefore, the HT-CO2 insufflation significantly suppressed intraperitoneal tumor spread of colorectal cancer, which suggested that HT-CO2 could prevent peritoneal dissemination during laparoscopic colorectal cancer surgery.

### HT-CO2 prevents port-site metastasis

**Fig 3:**
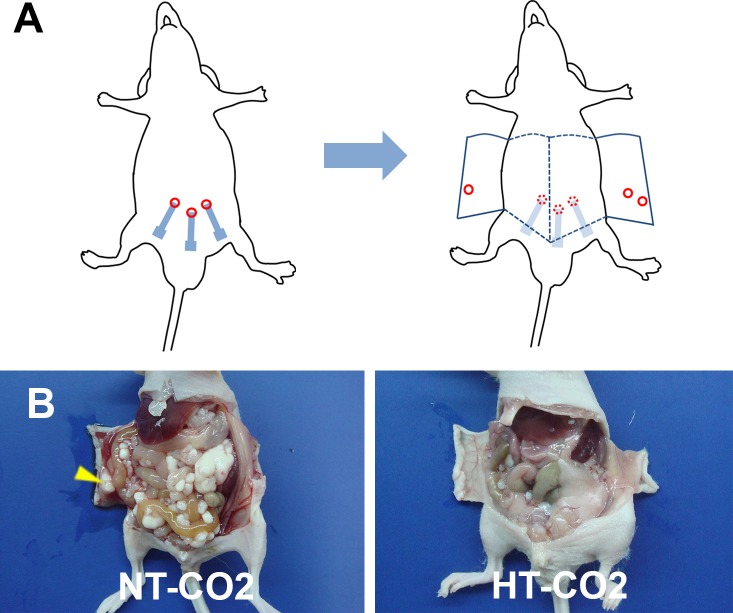
HT-CO2 significantly decreases port site metastasis. (**A**) Illustration of experimental setup. The location of port sites are recorded and marked. At 28 days after HT-CO2 or NT-CO2 insufflation, the marked port sites are checked for metastatic nodules. Laparotomy is performed and the port site metastases are confirmed from inside. (**B**) Port site metastasis forms in mice treated with NT-CO2 (arrows). The incidence of port site metastasis in NT-CO2 is 12.5% (3/24). In contrast, no port site metastases are detected in the HT-CO2 group.

The port sites were also examined specifically for macroscopic evidence of tumor implantation. No port site metastases were detected in the HT-CO2 group. In contrast, the incidence of port site metastasis in NT-CO2 was 12.5% (3/24) (Fig 3). HT-CO2 significantly reduced port site metastasis, suggesting that HT-CO2 can prevent port site metastases formation after laparoscopic colorectal cancer surgery.

### HT-CO2 causes slight, transient and recoverable peritoneal damages

**Fig 4:**
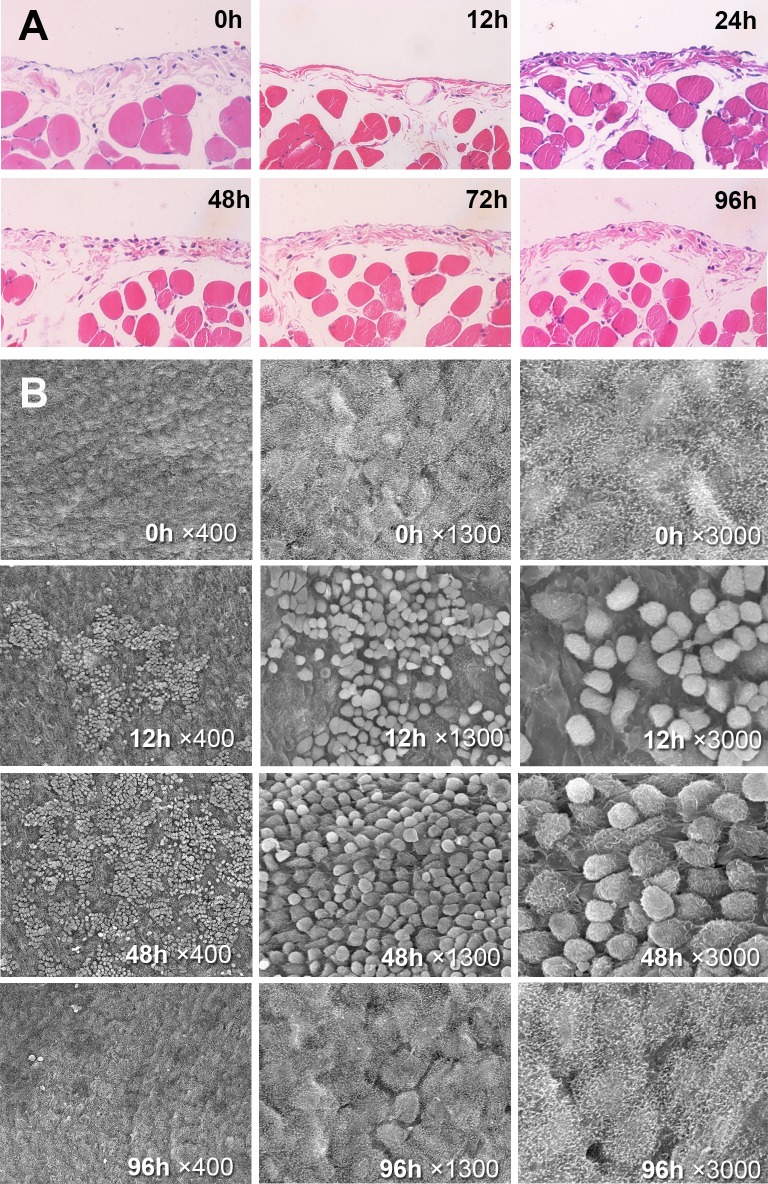
HT-CO2 causes slight, transient and recoverable peritoneal damages. (A) Light microscopic analysis of peritoneal alterations (from left to right, 0, 12, 24, 48, 72, 96h after HT-CO2 insufflation). The mesothelial cells detach and denuded basal lamina is apparently observed at 12 hours after HT-CO2 insufflations; (B) Scanning electron microscopic analysis of peritoneal alterations (from left to right, 0, 12, 48, 96h after HT-CO2 insufflation). The mesothelial cells detach and massive desquamations can be seen at 12 hours after HT-CO2 insufflations. However, the peritoneum recovers from 48 hours and restores to normal 96 hours later.

HT-CO2 was postulated to be harmless to normal tissues as hyperthermia at 43°C has been demonstrated to be cytotoxic to tumor cells but not normal tissues. However, the potential damage to peritoneum and peritoneal organs could not be excluded. We then evaluated the safety of the HT-CO2. All mice tolerated the HT-CO2 insufflation (43°C for 3h) well. Light microscopic and scanning electron microscopic analysis of the peritoneum after a postoperative 6h to 96h showed that HT-CO2 caused slight, transient and recoverable peritoneal damage (Fig 4). Histological findings at 6h after HT-CO2 insufflation treatment showed massive desquamation of mesothelial cells (Fig 4). Extensive detachment of mesothelial cells and denuded basal lamina were seen. However, the damaged peritoneum started to recover 48 hours later. The mesothelial cells returned to normal and regenerated to cover the denuded area. At 96 hours after HT-CO2 insufflations, most of the damaged are of the peritoneum recovered and returned to a normal morphology (Fig 4).

**Fig 5:**
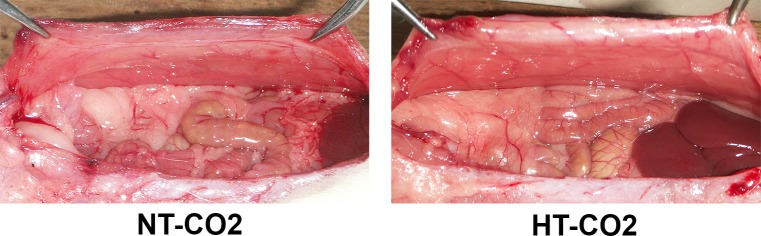
Peritoneal cavity of mice 4 weeks after HT-CO2 or NT-CO2 insufflation. HT-CO2 does not result in intra-abdominal adhesions, ascites, peritonitis or delayed healing of the port sites.

Peritoneal damage may result in or facilitate formation of peritoneal adhesions. The peritoneal cavity of the mice was examined at 4 weeks after the procedures were carried out. HT-CO2 caused no adhesion formation. Additionally, none of the animals showed formation of ascites, other signs of peritonitis or delayed healing of the port sites (Fig 5).

## Discussion

In this study, we examined a newly developed approach (hyperthermic CO2 insufflation, HT-CO2) for preventing peritoneal dissemination in laparoscopic surgery. Our data showed that HT-CO2 insufflation could significantly suppress peritoneal dissemination of colon cancer cells. Meanwhile, HT-CO2 caused only transient and recoverable peritoneal damage. These results suggest that HT-CO2 may serve as a novel modality for preventing peritoneal dissemination of colorectal cancer in laparoscopic surgery.

Peritoneal dissemination is one of the most frequent modes of recurrence after surgical resection of advanced gastrointestinal cancer [9]. It is associated with poor prognosis as treatment of peritoneal carcinomatosis is relatively ineffective and associated with high morbidity [9,10]. Therefore, the prevention of peritoneal dissemination is extremely important. Currently, laparoscopic surgery is increasingly used for advanced gastrointestinal cancers [11–13]. The risk of intraperitoneal tumor seeding as well as port site metastasis rises remarkably during laparoscopic surgery for these diseases. Surgical removal of advanced primary tumors unavoidably causes tissue wounding and cancer cells spillage, even though laparoscopic surgery is carried out. It has been demonstrated that peritoneal carcinomatosis can result from dissemination of cancer cells from the trauma of cancer surgery [14]. During laparoscopic surgery of advanced colorectal cancer, cancer cells may be released from the surface of the tumor. The CO2 may disseminate them to form peritoneal metastasis rather than local recurrence. Zayyan et al [15] reported that the rapid flow of CO2 can disseminate free cancer cells, contributing to intraperitoneal dispersal. The spillage or liberation of cells from the primary tumor is the critical event in tumor implantation [1]. Once this event has occurred, peritoneal and abdominal wall wounds are at risk for tumor formation. An effective approach for killing the spilled tumor cells and therefore reducing the likelihood of peritoneal dissemination is urgently needed, especially in patients with a high risk of postoperative intraperitoneal tumor spread. However, efficient methods following laparoscopic cancer surgery are still lacking.

In our previous studies we attempted a new approach (hyperthermic CO2 insufflation, HT-CO2). The CO2 used for pneumoperitoneum establishment was heated to hyperthermic temperature (43–44°C) to kill the spilled colorectal cancer cells generated during laparoscopic cancer surgery and therefore prevent peritoneal dissemination. In vitro studies showed that HT-CO2 has significant cytotoxic effect on colon and gastric cancer cells [7,8], suggesting that HT-CO2 is an attractive solution for preventing peritoneal metastases during laparoscopic surgery of gastrointestinal cancer. In this study we examined its effectiveness through an in vivo peritoneal dissemination model. The current in vivo study showed that HT-CO2 insufflation could significantly decrease intraperitoneal dissemination of spilled colon cancer cells and local peritoneal carcinomatosis. Additionally, HT-CO2 could significantly inhibit the formation of port site metastases. This is the first report showing that HT-CO2 can suppress peritoneal dissemination of colon cancer cells. Hyperthermia has been demonstrated to be effective for cancer treatments [16]. In contrast with hyperthermia alone, HT-CO2 can provides a much stronger and satisfactory cytotoxic effect [7,8]. Peritoneal dissemination of tumor cells is thought to involve several sequential steps: exfoliation or spillage, adhesion of dissociated tumor cells to the peritoneum, invasion of the tissue by degrading basement membrane, and proliferation around the vessels by angiogenesis [17]. Ex-vivo studies of the adhesion of cancer cells to the peritoneal surface show that the detached cells can rapidly adhere to the peritoneal wall. The first adhesion occurs within 80min through the microvilli and 76% of the inoculated cells are fixed within 24h of the inoculation [18,19]. HT-CO2 may directly kill the spilled cancer cells as soon as the cells detach from the primary tumor and inhibit the initial adherence of cells to the peritoneal mesothelium. However, the underlying mechanism needs further investigation. Our data also showed that HT-CO2 is safe enough for application. HT-CO2 was well tolerated and caused only slight, transient and recoverable peritoneal damage. The peritoneal damage resulting from the HT-CO2 was even less severe than that of NT-CO2. This is consistent with the well-established effect of hyperthermia in that hyperthermia has a selective cytotoxic effect on cancer cells but not normal cells. Normal human tissues and cells can tolerate hyperthermia at 43–44°C well.

Therapeutic approaches for prevention of peritoneal dissemination are few, especially for laparoscopic gastrointestinal cancer surgery. Simple irrigation with distilled water or saline solution has been shown to be ineffective in removing tumor cells and preventing local recurrences [20]. In the last decade, intraperitoneal chemotherapy and hyperthermic intraperitoneal chemotherapy (HIPEC) have been shown to be effective for treatment of peritoneal carcinomatosis [10,21]. In selected patients with peritoneal carcinomatosis from colon cancer, HIPEC shows improved survival [10]. However, HIPEC can result in serious peritoneal damage and causes severe side effects [22]. The HIPEC+cytoreductive surgery (CRS) is reported to be associated with high morbidity and mortality (3.0% and 23.8%, respectively) [22]. Patient selection for HIPEC and CRS is critical and can only be used with a very limited group. Recently, Sloothaak et al [23] attempted adjuvant laparoscopic hyperthermic intraperitoneal chemotherapy (LHIPEC) for patients at risk of peritoneal carcinomatosis of colorectal cancer. Jung et al. [24] developed a device to apply hyperthermic pressurized intraperitoneal aerosol chemotherapy (H-PAC) and reported that H-PAC is feasible and safe in a porcine model. However, the efficiency and long-term outcome of LHIPEC and H-PAC are unknown. In this study, we did not compare HIPEC, LHIPEC and H-PAC with HT-CO2 as the devices used for mouse models are not available. Our study showed that HT-CO2 can effectively suppress peritoneal dissemination. It provides an attractive solution for peritoneal dissemination in laparoscopic cancer surgery. The spilled cancer cells can be killed as soon as they are generated following laparoscopic surgery. Additionally, HT-CO2 only causes minor peritoneal damage and so postoperative complications will be minimized. One potential advantage of intraperitoneal HT-CO2 is that it can be used in all patients at risk of peritoneal metastasis. HT-CO2 therefore serves as a promising adjuvant therapy for prevention of peritoneal dissemination in laparoscopic advanced gastrointestinal cancer surgery.

A limitation of this study is that a murine cell inoculation model is used. The colon cancer cells are injected into the peritoneal cavity prior to the test procedure to mimic the spillage of tumor cells during surgery. Thus far, the vast majority of the published animal studies concerning peritoneal carcinomatosis and/or port site metastasis have utilized the cell inoculation model [25,26]. This model allows assessment of the spread of free tumor cells within the abdomen. However, it is less than ideal for the investigation of peritoneal dissemination. The injection of colon cancer cells may not fully simulate the liberation of cancer cells from the primary tumor during laparoscopic colorectal cancer surgery since primary tumors are present prior to surgery in the clinical setting. Because of the limitations of the cell inoculation model, we attempted to establish a mouse model of cecal cancer with serosal invasion using the methods previously described in the pilot study [27]. However, the model was not stable and has unacceptable bias because it is difficult to establish tumors of identical size. It was therefore not used in this study. Furthermore, the murine model does not allow laparoscopic manipulation of tumors in the abdomen. Further investigations in large animal models with greater similarity to a clinical setting are required.

In conclusion, the new HT-CO2 approach can significantly suppress peritoneal dissemination and port-site metastases of colon cancer cells while only causing slight, transient and recoverable peritoneal damage. HT-CO2 may serve as a novel adjuvant therapy for prevention of peritoneal dissemination during laparoscopic surgery of advanced colorectal cancer.

## Supporting information

S1 File The post-operative care after HT-CO2 or NT-CO2 insufflation. (DOC)

## References

[1] Lee SW, Southall J, Allendorf J, Bessler M, Whelan RL. Traumatic handling of the tumor independent of pneumoperitoneum increases port site implantation rate of colon cancer in a murine model. Surgical endoscopy. 1998;12(6):828–34. Epub 1998/06/05. 9602000 10.1007/s004649900723

[2] Zayyan KS, Christie-Brown JS, Noorden S, Yiu CY, Sellu DP, Mathie RT. Rapid flow carbon dioxide laparoscopy disperses cancer cells into the peritoneal cavity but not the port sites in a new rat model. Surgical Endoscopy And Other Interventional Techniques. 2003;17(2):273–7. 10.1007/s00464-002-8824-8 12399832

[3] Volz J, Koster S, Spacek Z, Paweletz N. The influence of pneumoperitoneum used in laparoscopic surgery on an intraabdominal tumor growth. Cancer. 1999;86(5):770–4. Epub 1999/08/27. 10463974

[4] Peng Y, Zheng M, Ye Q, Chen X, Yu B, Liu B. Heated and humidified CO2 prevents hypothermia, peritoneal injury, and intra-abdominal adhesions during prolonged laparoscopic insufflations. The Journal of surgical research. 2009;151(1):40–7. Epub 2008/07/22. 10.1016/j.jss.2008.03.039 18639246

[5] Gupta A, Watson DI, Ellis T, Jamieson GG. Tumour implantation following laparoscopy using different insufflation gases. ANZ journal of surgery. 2002;72(4):254–7. Epub 2002/05/02. 11982509 10.1046/j.1445-2197.2002.02385.x

[6] Pestieau S, Sugarbaker P. Treatment of primary colon cancer with peritoneal carcinomatosis. Diseases of the Colon & Rectum. 2000;43(10):1341–6. 11052509 10.1007/BF02236627

[7] Peng Y, Zheng M, Feng B, Chen X, Yu B, Lu A, et al Hyperthermic CO2 pneumoperitoneum induces apoptosis in human colon cancer cells through Bax-associated mitochondrial pathway. Oncology reports. 2008;19(1):73–9. Epub 2007/12/22. 18097578

[8] Zhou HM, Feng B, Zhao HC, Zheng MH. Antitumor effects of hyperthermic CO2 pneumoperitoneum on human gastric cancer cells. Asian Pacific journal of cancer prevention: APJCP. 2012;13(1):117–22. Epub 2012/04/17. 22502652 10.7314/apjcp.2012.13.1.117

[9] Marz L, Piso P. Treatment of peritoneal metastases from colorectal cancer. Gastroenterology report. 2015;3(4):298–302. Epub 2015/10/02. PubMed Central PMCID: PMCPMC4650975. 10.1093/gastro/gov044 26424828 PMC4650975

[10] Sloothaak DA, Mirck B, Punt CJ, Bemelman WA, van der Bilt JD, D'Hoore A, et al Intraperitoneal chemotherapy as adjuvant treatment to prevent peritoneal carcinomatosis of colorectal cancer origin: a systematic review. British journal of cancer. 2014;111(6):1112–21. Epub 2014/07/16. PubMed Central PMCID: PMCPMC4453838. 10.1038/bjc.2014.369 25025964 PMC4453838

[11] Akiyoshi T. Technical feasibility of laparoscopic extended surgery beyond total mesorectal excision for primary or recurrent rectal cancer. World journal of gastroenterology: WJG. 2016;22(2):718–26. Epub 2016/01/27. PubMed Central PMCID: PMCPMC4716071. 10.3748/wjg.v22.i2.718 26811619 PMC4716071

[12] Kim IY, Kim BR, Kim HS, Kim YW. Differences in clinical features between laparoscopy and open resection for primary tumor in patients with stage IV colorectal cancer. OncoTargets and therapy. 2015;8:3441–8. Epub 2015/12/08. PubMed Central PMCID: PMCPMC4657796. 10.2147/OTT.S93420 26640384 PMC4657796

[13] Barchi LC, Jacob CE, Bresciani CJ, Yagi OK, Mucerino DR, Lopasso FP, et al MINIMALLY INVASIVE SURGERY FOR GASTRIC CANCER: TIME TO CHANGE THE PARADIGM. Arquivos brasileiros de cirurgia digestiva: ABCD = Brazilian archives of digestive surgery. 2016;29(2):117–20. Epub 2016/07/22. PubMed Central PMCID: PMCPMC4944749. 10.1590/0102-6720201600020013 27438040 PMC4944749

[14] Sugarbaker PH. Strategies for the prevention and treatment of peritoneal carcinomatosis from gastrointestinal cancer. Cancer investigation. 2005;23(2):155–72. Epub 2005/04/09. 15813509

[15] Zayyan KS, Christie-Brown JS, Van Noorden S, Yiu CY, Sellu DP, Mathie RT. Rapid flow carbon dioxide laparoscopy disperses cancer cells into the peritoneal cavity but not the port sites in a new rat model. Surgical endoscopy. 2003;17(2):273–7. Epub 2002/10/26. 10.1007/s00464-002-8824-8 12399832

[16] Hildebrandt B, Wust P, Ahlers O, Dieing A, Sreenivasa G, Kerner T, et al The cellular and molecular basis of hyperthermia. Critical reviews in oncology/hematology. 2002;43(1):33–56. Epub 2002/07/06. 12098606 10.1016/s1040-8428(01)00179-2

[17] Aoyagi T, Terracina KP, Raza A, Takabe K. Current treatment options for colon cancer peritoneal carcinomatosis. World journal of gastroenterology: WJG. 2014;20(35):12493–500. Epub 2014/09/26. PubMed Central PMCID: PMCPMC4168082. 10.3748/wjg.v20.i35.12493 25253949 PMC4168082

[18] Asao T, Yazawa S, Kudo S, Takenoshita S, Nagamachi Y. A novel ex vivo method for assaying adhesion of cancer cells to the peritoneum. Cancer letters. 1994;78(1–3):57–62. Epub 1994/04/01. 8180969 10.1016/0304-3835(94)90031-0

[19] Kudo M, Asao T, Hashimoto S, Kuwano H. Closed continuous hyperthermic peritoneal perfusion model in mice with peritoneal dissemination of colon 26. International journal of hyperthermia: the official journal of European Society for Hyperthermic Oncology, North American Hyperthermia Group. 2004;20(4):441–50. Epub 2004/06/19. 10.1080/02656730310001637352 15204523

[20] Roviello F, Caruso S, Neri A, Marrelli D. Treatment and prevention of peritoneal carcinomatosis from gastric cancer by cytoreductive surgery and hyperthermic intraperitoneal chemotherapy: overview and rationale. European journal of surgical oncology: the journal of the European Society of Surgical Oncology and the British Association of Surgical Oncology. 2013;39(12):1309–16. Epub 2013/11/05. 10.1016/j.ejso.2013.10.010 24183797

[21] Loggie BW, Thomas P. Gastrointestinal Cancers With Peritoneal Carcinomatosis: Surgery and Hyperthermic Intraperitoneal Chemotherapy. Oncology (Williston Park, NY). 2015;29(7):515–21. Epub 2015/07/17. 26178339

[22] Baratti D, Kusamura S, Iusco D, Bonomi S, Grassi A, Virzi S, et al Postoperative complications after cytoreductive surgery and hyperthermic intraperitoneal chemotherapy affect long-term outcome of patients with peritoneal metastases from colorectal cancer: a two-center study of 101 patients. Diseases of the colon and rectum. 2014;57(7):858–68. Epub 2014/06/06. 10.1097/DCR.0000000000000149 24901687

[23] Sloothaak DA, Gardenbroek TJ, Crezee J, Bemelman WA, Punt CJ, Buskens CJ, et al Feasibility of adjuvant laparoscopic hyperthermic intraperitoneal chemotherapy in a short stay setting in patients with colorectal cancer at high risk of peritoneal carcinomatosis. European journal of surgical oncology: the journal of the European Society of Surgical Oncology and the British Association of Surgical Oncology. 2014;40(11):1453–8. Epub 2014/07/31. 10.1016/j.ejso.2014.06.012 25073662

[24] Jung DH, Son SY, Oo AM, Park YS, Shin DJ, Ahn SH, et al Feasibility of hyperthermic pressurized intraperitoneal aerosol chemotherapy in a porcine model. Surgical endoscopy. 2015. Epub 2015/12/31. 10.1007/s00464-015-4738-0 26715024

[25] Hirabayashi Y, Yamaguchi K, Shiraishi N, Adachi Y, Kitamura H, Kitano S. Development of port-site metastasis after pneumoperitoneum. Surgical endoscopy. 2002;16(5):864–8. Epub 2002/05/09. 10.1007/s00464-001-9121-7 11997839

[26] Mutter D, Hajri A, Tassetti V, Solis-Caxaj C, Aprahamian M, Marescaux J. Increased tumor growth and spread after laparoscopy vs laparotomy: influence of tumor manipulation in a rat model. Surgical endoscopy. 1999;13(4):365–70. Epub 1999/03/27. 10094749 10.1007/s004649900991

[27] Takeuchi H, Inomata M, Fujii K, Ishibashi S, Shiraishi N, Kitano S. Increased peritoneal dissemination after laparotomy versus pneumoperitoneum in a mouse cecal cancer model. Surgical endoscopy. 2004;18(12):1795–9. Epub 2005/04/06. 10.1007/s00464-003-9322-3 15809793

