# Supplementary material for: Inhibition of peritoneal dissemination of colon cancer by hyperthermic CO2 insufflation: A novel approach to prevent intraperitoneal tumor spread
Source: PLoS One. 2017 Feb 16;12(2):e0172097. doi: 10.1371/journal.pone.0172097 (PMC5313196; doi:10.1371/journal.pone.0172097)
Supplement: S1 File — (DOC) [file pone.0172097.s001.doc]

The post-operative care received by the animals was conducted as described below. All mice were recovered in a specific pathogen-free barrier facility. The mice were recovered in a clean bedded cage placed over a heating pad in a warming environment. The mice were covered with a drape. Respiration rate and depth were checked every 10 to 15 minutes, until they had recovered their balance and were able to remain upright in a sternal position. The mice were then placed back into their cages after they had fully recovered from anesthesia. Post-operative analgesics were used for all mice undergoing surgery (Bupivicaine + acetaminophen-treated drinking water for 48 hours post-surgically). Bupivicaine is applied topically at the incision site (2 drops 0.25% Bupivicaine at the trocar site). The acetaminophen-treated drinking water (1.6mg/ml) was prepared by adding 12.5 ml Children’s Tylenol Elixir (32 mg/ml Acetaminophen) to 237.5 ml of drinking water. Fresh acetaminophen solution was prepared every day. Upon observing signs of pain or discomfort in the mice, analgesic dosing and administration was adjusted to minimize pain. All mice were observed twice a day after the procedure. The observations were recorded and parameters such as appetite and wound healing were assessed. No adverse signs (hunched posture, dehydration, labored breathing, decreased activity, rough hair coat, porphyrin staining, or weight-loss) were observed after surgery.
